# Dapagliflozin in Chronic Kidney Disease: Insights from Network Pharmacology and Molecular Docking Simulation

**DOI:** 10.3390/life15030437

**Published:** 2025-03-11

**Authors:** Atthaphong Phongphithakchai, Aman Tedasen, Ratana Netphakdee, Rattana Leelawattana, Thatsaphan Srithongkul, Sukit Raksasuk, Jason C. Huang, Moragot Chatatikun

**Affiliations:** 1 Nephrology Unit, Division of Internal Medicine, Faculty of Medicine, Prince of Songkla University, Songkhla 90110, Thailand; ton331@hotmail.com; 2 Department of Medical Technology, School of Allied Health Sciences, Walailak University, Nakhon Si Thammarat 80160, Thailand; aman.te@wu.ac.th (A.T.); ratana.ne@mail.wu.ac.th (R.N.); 3 Research Excellence Center for Innovation and Health Products (RECIHP), Walailak University, Nakhon Si Thammarat 80160, Thailand; 4 Endocrinology and Metabolism Unit, Division of Internal Medicine, Faculty of Medicine, Prince of Songkla University, Songkhla 90110, Thailand; lrattana@medicine.psu.ac.th; 5 Division of Nephrology, Department of Medicine, Faculty of Medicine, Siriraj Hospital, Mahidol University, Bangkok 10700, Thailand; thatsaphan@gmail.com (T.S.); kobsukit@gmail.com (S.R.); 6 Department of Biotechnology and Laboratory Science in Medicine, National Yang Ming Chiao Tung University, Taipei 112304, Taiwan; jasonhuang@nycu.edu.tw

**Keywords:** dapagliflozin, chronic kidney disease, network pharmacology, molecular docking, protein-protein interaction

## Abstract

Chronic kidney disease (CKD) involves inflammation, oxidative stress, and fibrosis, leading to renal dysfunction. Dapagliflozin, an SGLT2 inhibitor, shows renoprotective effects beyond glucose control, but its precise molecular mechanisms remain unclear. This study utilizes network pharmacology and molecular docking to elucidate its multi-target effects in CKD. Dapagliflozin’s SMILES structure was analyzed for ADMET properties. Potential targets were identified via SwissTargetPrediction, GeneCards, and SEA, and common CKD-related targets were determined. A protein–protein interaction (PPI) network was constructed, and key pathways were identified using GO and KEGG enrichment analyses. Molecular docking was conducted to validate dapagliflozin’s binding affinities with hub proteins. A total of 208 common targets were identified, including EGFR, GSK3β, and IL-6. GO and KEGG analyses highlighted key pathways, such as PI3K-Akt, MAPK, and AGE-RAGE, involved in inflammation, oxidative stress, and metabolic regulation. Molecular docking confirmed strong binding affinities with EGFR (−8.42 kcal/mol), GSK3β (−7.70 kcal/mol), and IL-6 (−6.83 kcal/mol). Dapagliflozin exhibits multi-target therapeutic potential in CKD by modulating inflammation, oxidative stress, and metabolic pathways. This integrative approach enhances the understanding of its mechanisms, supporting future experimental validation and clinical application in CKD management.

## 1. Introduction

Chronic kidney disease (CKD) is a significant global health issue that affects millions of individuals and imposes substantial economic and social costs [1]. With a prevalence of approximately 9.5% across 162 countries, CKD strains healthcare systems and contributes to elevated morbidity, mortality, and diminished quality of life [2]. The pathophysiology of CKD is multifaceted, involving mechanisms such as glomerular hypertension, tubulointerstitial fibrosis, and inflammation [3,4]. These processes cumulatively lead to a progressive decline in renal function and eventual end-stage renal disease (ESRD), necessitating interventions such as dialysis or transplantation [5]. Inflammation plays a particularly critical role in CKD progression, marked by elevated levels of pro-inflammatory cytokines, such as interleukin-6 (IL-6) and tumor necrosis factor-α (TNF-α) [6,7]. Additionally, oxidative stress and mitochondrial dysfunction exacerbate renal damage, highlighting the importance of addressing these mechanisms in therapeutic development [8,9].

Dapagliflozin, an SGLT2 inhibitor, has shown promise as a therapeutic agent for CKD [10]. By inhibiting glucose reabsorption in the proximal tubules, dapagliflozin induces glucosuria, reducing blood glucose levels [11]. Beyond its glucose-lowering effects, dapagliflozin demonstrates renoprotective mechanisms, such as reducing intraglomerular pressure, lowering albuminuria, and attenuating tubulointerstitial fibrosis [12,13]. Preclinical studies in animal models have supported its ability to reduce markers of renal injury and improve renal outcomes [14,15]. Clinical trials have further established its efficacy in reducing ESRD progression and cardiovascular events in CKD patients, regardless of diabetes status [16,17]. However, the molecular mechanisms underlying these effects remain partially understood and warrant further investigation.

Network pharmacology, a systems biology approach, provides a powerful framework for elucidating the complex interactions between drugs, targets, and disease pathways. By integrating data from multiple databases and computational tools, this approach enables the identification of potential molecular targets and pathways involved in drug action [18]. This approach enables a comprehensive analysis of the interactions between drugs, targets, and disease pathways, offering insights into potential therapeutic mechanisms and novel targets. The application of network pharmacology to dapagliflozin provides an opportunity to uncover its multi-target effects and explore its broader therapeutic potential in CKD.

This study aims to utilize network pharmacology and molecular docking to investigate the mechanisms of dapagliflozin in CKD. By integrating data from diverse databases, we systematically identify and analyze the common molecular targets of dapagliflozin and CKD, construct protein–protein interaction (PPI) networks, and perform gene ontology (GO) and KEGG pathway enrichment analyses. Additionally, molecular docking simulations are conducted to validate the binding interactions of dapagliflozin with key hub proteins. These comprehensive analyses aim to provide novel insights into dapagliflozin’s therapeutic mechanisms, laying the groundwork for future experimental and clinical validation.

## 2. Materials and Methods

### 2.1. Drug Activity Evaluation of Dapagliflozin

The drug activity of dapagliflozin was evaluated using in silico ADMET (Absorption, Distribution, Metabolism, Excretion, and Toxicity) analysis and drug-likeness prediction through the SwissADME (http://www.swissadme.ch/ accessed on 2 November 2024) and pkCSM platforms (https://biosig.lab.uq.edu.au/pkcsm/ accessed on 2 November 2024). Dapagliflozin’s adherence to Lipinski’s rule of five was assessed to evaluate its potential as an orally bioavailable drug. The analyses included physicochemical property evaluation, lipophilicity and solubility prediction, pharmacokinetic profiling, and toxicity assessment, which were generated using both SwissADME and pkCSM [19].

### 2.2. Screening the Targets of Dapagliflozin and Chronic Kidney Disease

The potential targets of dapagliflozin were gathered from the SwissTargetPrediction (http://www.swisstargetprediction.ch/ accessed on 4 November 2024), Super-PRED (https://prediction.charite.de/subpages/target_prediction.php/ accessed on 4 November 2024), and similarity ensemble approach or SEA (https://sea.bkslab.org/ accessed on 4 November 2024) [20]. The names of target proteins were translated into gene names in the UniProt (http://www.uniprot.org/ accessed on 5 November 2024) database [21]. The CKD-related genes were derived from the GeneCards (https://www.GeneCards.org/ accessed on 7 November 2024) databases by searching the keywords of chronic kidney disease [22].

### 2.3. Construction of Drug-Target Network

The common targets of dapagliflozin and CKD were determined using a Venn diagram (https://bioinfogp.cnb.csic.es/tools/venny/index.html accessed on 10 November 2024) [23]. Intersecting genes were the potential targets of dapagliflozin, and they overlapped with CKD.

### 2.4. Construction of Protein-Protein Interaction (PPI) Network

Intersecting genes from a Venn diagram were uploaded to the STRING database (https://string-db.org/ accessed on 12 November 2024), and *Homo sapiens* was chosen for species [24]. The result from the STRING database was downloaded and imported to Cytoscape 3.8.0 software (https://cytoscape.org/ accessed on 13 November 2024) [25]. Then the protein interaction information was obtained, a network analysis of the PPI network was performed, and the top 10 targets of the degree centrality (DC) values were screened.

### 2.5. Gene Functions and Pathway Enrichment Analysis with Potential Targets

Gene ontology (GO) enrichment analysis and the Kyoto Encyclopedia of Genes and Genomes (KEGG) pathway analyses were carried out using ShinyGO 0.80 (http://bioinformatics.sdstate.edu/go/ accessed on 15 November 2024) [26]. The GO enrichment and KEGG pathway analyses were screened for *p* < 0.05. The top 20 results from the GO enrichment and KEGG pathway analyses were presented. The GO enrichment analysis mainly shows three aspects of biology, such as biological process, molecular component, and cellular component. It is widely used for gene function classification and target function distribution prediction.

### 2.6. Protein and Compound Preparation

To enhance our understanding of the relationship, modes of interaction, and mechanisms of action between the candidate proteins (or hub targets) and dapagliflozin, we employed a computational technique known as molecular docking. This simulation methodology enables the prediction of the affinity and orientation of a compound within a protein’s structure, thereby providing insights into potential binding modes. To initiate this process, we obtained the 3D structures of the pivotal proteins from the Protein Data Bank (PDB) and performed essential cleaning procedures to eliminate water molecules or other small molecules that might interfere with the docking process [27]. This preparatory phase was conducted using BIOVIA Discovery Studio software (Version 2021 Client). The 3D protein structures of the top 10 target proteins, including GAPDH (PDB 6M61), IL-6 (PDB 1ALU), SRC (PDB 1Y57), EGFR (PDB 1M17), HSP90AA1 (PDB 4AWQ), NFKB (PDB 3GUT), CASP3 (PDB 3KJF), HSP90AB1 (PDB 5UCJ), MAPK3 (PDB 4QTB), and GSK3β (PDB 6TCU), were downloaded from the RCSB Protein Data Bank (https://www.rcsb.org/ accessed on 20 November 2024) in PDB format [28]. Details of the proteins were collected from the PDB database (Table 1). The Protein Data Bank (PDB) is a global repository of 3D structural data for major proteins. The co-crystallized ligand and water molecules were removed from the protein structures. Polar hydrogens, charges, solvation parameters, and fragmental volumes were assigned to the protein using the Kollman united atom force field via AutoDock Tools (ADT) (Version 1.1.2) [29]. The cleaned protein structures were then saved in the PDBQT file format.

The three-dimensional (3D) structure of dapagliflozin (CID 9887712) was retrieved from the PubChem database (http://pubchem.ncbi.nlm.nih.gov/ accessed on 25 November 2024) and prepared for docking. Known tyrosinase inhibitors or positive controls were also retrieved from co-crystallized protein structures. This preparation involved structural optimization, the addition of missing hydrogen atoms, and charge correction. The ligand was further optimized and converted to a PDB file using UCSF Chimera software (version 1.17.1) [30]. Energy minimization using conjugate and steepest descent methods, along with the addition of charges for correcting ionization, was employed to prepare the ligands. Missing hydrogen atoms and polar hydrogens were added to all ligands at a pH of 7.4. Bond orders, angles, and topology were assigned to optimize the structure. AutoDock Tools (ADT) automatically assigned Gasteiger charges and default atomic parameters. Finally, the optimized structures were saved in PDBQT file format using ADT.

### 2.7. Molecular Docking Simulation

Molecular docking was performed using AutoDock version 4.2. The Lamarckian genetic algorithm method was employed for the molecular docking experiment, and it was conducted with AutoDock4 software. During the procedure, the protein structure was treated as a rigid molecule, while the ligand was considered flexible. Default parameter values in AutoDockTools (ADT) were used for all other settings. For conformational sampling, fifty genetic algorithm (GA) runs were executed with a population size of 200. A docking box was constructed to completely encompass the binding site of the receptor protein. The parameters of the docking box were recorded (Table 1). Molecular docking was carried out using AutoDockTools 1.5.7, where the binding energy magnitude was used to assess the likelihood of binding between the receptor and the ligand. The optimal pose was determined as the conformation with the lowest binding energy (kcal/mol). The interactions of natural ligands or drugs were compared to the best-docked pose of dapagliflozin. Binding energy data from the molecular docking process were gathered using AutoDockTools 1.5.7. Finally, potential protein–ligand interactions and binding modes were analyzed and visualized using the BIOVIA Discovery Studio Visualizer software (Accelrys, San Diego, CA, USA) [19].

## 3. Results

### 3.1. Drug Activity of Dapagliflozin

The drug activity of dapagliflozin was evaluated using ADMET analysis and drug-likeness prediction through SwissADME and pkCSM platforms. As shown in Table S1, the analysis revealed key physicochemical properties, pharmacokinetics, and toxicity profiles. The molecular weight (MW) of dapagliflozin is 408.87 g/mol, with a formula of C_21_H_25_ClO_6_. It exhibits six hydrogen bond acceptors, four hydrogen bond donors, and a topological polar surface area (TPSA) of 99.38 Å^2^. Its predicted lipophilicity (LogP) values from different models (iLOGP, XLOGP3, WLOGP, Silicos-IT Log P) range from 1.07 to 3.17, with a consensus LogP of 2.18, indicating moderate lipophilicity. Dapagliflozin is classified as soluble based on ESOL predictions and moderately soluble by aliphatic and silicos-IT classifications.

In terms of pharmacokinetics, dapagliflozin shows high gastrointestinal (GI) absorption but does not cross the blood–brain barrier (BBB). It is a substrate for P-glycoprotein but not for CYP2D6 or CYP3A4 enzymes. It inhibits CYP2D6 but does not inhibit other key cytochrome P450 enzymes (CYP1A2, CYP2C19, CYP2C9, or CYP3A4). The predicted skin permeability (log Kp) is −7.13 cm/s, and the total clearance is 0.194 log mL/min/kg.

From a toxicity perspective, dapagliflozin demonstrates no AMES toxicity, hepatotoxicity, or skin sensitization. It is not a substrate or inhibitor of hERG channels, indicating low cardiac risk. Predicted oral rat acute toxicity (LD50) is 2.475 mol/kg, and chronic toxicity (LOAEL) is 3.63 log mg/kg/day. It also shows moderate environmental toxicity with a Tetrahymena pyriformis toxicity (log µg/L) of 0.289 and minnow toxicity (log mM) of 1.079.

Dapagliflozin adheres to Lipinski’s rule of five with zero violations, suggesting good oral bioavailability (bioavailability score of 0.55). No alerts for pan-assay interference compounds alerts (PAINS), Brenk, or lead-likeness were identified. The synthetic accessibility score is 4.52, indicating a moderately complex synthesis. These results highlight dapagliflozin’s favorable drug-likeness profile and suitability for oral administration, with minimal toxicity risks.

### 3.2. Targets of Dapagliflozin and Chronic Kidney Disease

The screening process identified a total of 231 potential targets for dapagliflozin through the SwissTargetPrediction, Super-PRED, and Similarity Ensemble Approach (SEA) platforms. These targets were converted into gene names using the UniProt database for standardization. Concurrently, 16,154 chronic kidney disease (CKD)-related genes were extracted from the GeneCards database using the keyword “chronic kidney disease”.

### 3.3. Prediction of Common Targets Between Dapagliflozin and Chronic Kidney Disease

**Figure 1:**
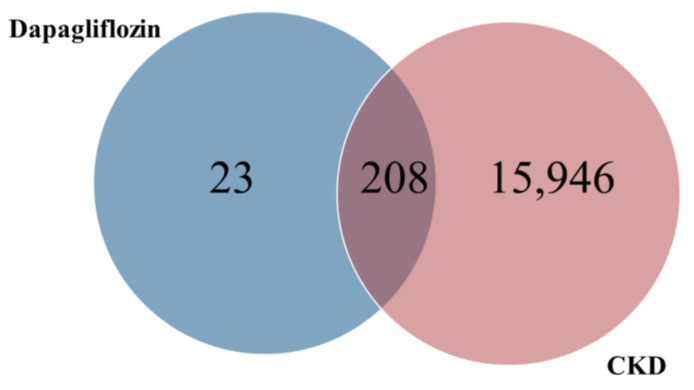
Overlapping targets of dapagliflozin and chronic kidney disease.

The common targets of dapagliflozin and CKD were identified using a Venn diagram analysis. Out of the 231 potential targets of dapagliflozin and 16,154 CKD-related genes, a total of 208 overlapping targets were identified, as shown in Figure 1 and Table 2. These overlapping genes represent the potential targets through which dapagliflozin may exert therapeutic effects on CKD. This result highlights a significant overlap and provides a foundation for further investigation into the molecular mechanisms underlying the drug’s action in CKD.

### 3.4. Construction of PPI Networks

**Figure 2:**
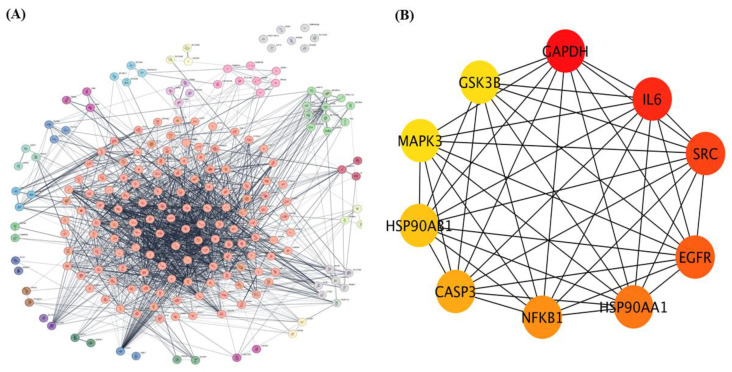
The protein–protein interaction (PPI) network of overlapping targets of dapagliflozin and chronic kidney disease. (**A**) protein-protein interaction (PPI) network of 208 overlapping genes identified as common targets of dapagliflozin and chronic kidney disease (CKD). The network was constructed using the STRING database with *Homo sapiens* as the selected species and visualized in Cytoscape 3.10.3. The complex network displays interactions among these genes, with edges representing experimentally and computationally determined connections. This method organizes key proteins into clusters based on the MCL cluster network in Cytoscape. (**B**) The top 10 hub genes from the PPI network, identified using the cytoHubba plugin in Cytoscape based on degree centrality (DC) values. The hub genes include GAPDH, IL-6, SRC, EGFR, HSP90AA1, NFKB1, CASP3, HSP90AB1, MAPK3, and GSK3B. Node coloration, ranging from yellow to dark red, indicates DC scores, with darker red nodes representing higher centrality and greater importance within the network. These hub genes are potential key players in the mechanism of action of dapagliflozin in CKD.

The 208 overlapping genes identified as common targets of dapagliflozin and CKD were uploaded to the STRING database to construct a protein–protein interaction (PPI) network, with *Homo sapiens* selected as the species, as shown in Figure 2A. The interaction data were exported and imported into Cytoscape 3.10.3 for network visualization and analysis.

Using the Cytoscape plugin cytoHubba, a network analysis was performed to identify the top 10 hub genes based on degree centrality (DC) values as shown in Figure 2B. These hub targets, which represent the most interconnected and potentially critical nodes in the PPI network, are glyceraldehyde-3-phosphate dehydrogenase (GAPDH), interleukin-6 (IL-6), proto-oncogene tyrosine-protein kinase Src (SRC), epidermal growth factor receptor (EGFR), heat shock protein HSP 90-alpha (HSP90AA1), nuclear factor NF-kappa-B p105 subunit (NFKB1), caspase-3 (CASP3), heat shock protein HSP 90-beta (HSP90AB1), mitogen-activated protein kinase 3 (MAPK3), and glycogen synthase kinase-3 beta (GSK3β). Their significance within the network is highlighted by node coloration, where darker and redder hues correspond to higher scores, indicating greater importance in the network. These hub targets may play pivotal roles in the mechanism of action of dapagliflozin in CKD.

### 3.5. KEGG Pathway Enrichment Analysis and GO Enrichment Analysis

GO Enrichment analysis and KEGG pathway enrichment analysis were conducted to explore the biological roles of the intersecting genes identified as targets of dapagliflozin in chronic kidney disease (CKD).

#### 3.5.1. KEGG Pathway Enrichment Analysis

**Figure 3:**
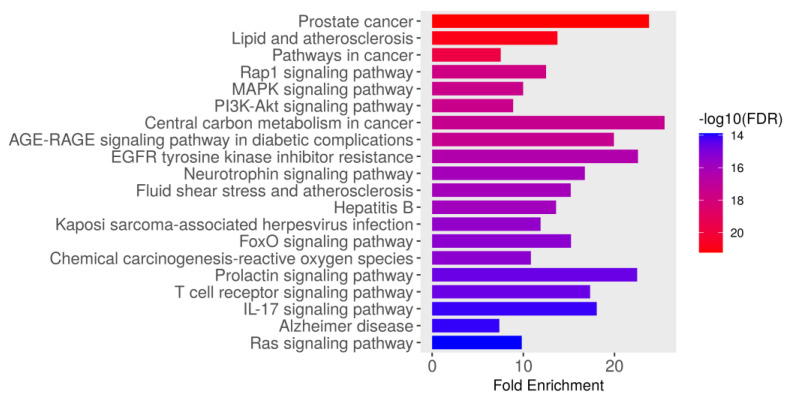
The Kyoto Encyclopedia of Genes and Genomes (KEGG) pathway enrichment analysis of genes associated with dapagliflozin and chronic kidney disease (CKD). The bar plot illustrates the top enriched pathways based on fold enrichment, ranked from most to least enriched. The color gradient represents the −log10(FDR) values, with red indicating the highest statistical significance and blue indicating lower significance.

The KEGG pathway enrichment analysis identified the top 20 significantly enriched pathways associated with the intersecting targets of dapagliflozin and CKD based on their fold enrichment and −log10(FDR) values, as shown in Figure 3 and Table 3. The −log10(FDR) scores indicate the statistical significance of each pathway, with higher values reflecting greater significance. Among these, the most enriched pathway was prostate cancer (hsa05215), followed by lipid and atherosclerosis (hsa05417), pathways in cancer (hsa05200), Rap1 signaling pathway (hsa04015), MAPK signaling pathway (hsa04010), PI3K-Akt signaling pathway (hsa04151), AGE-RAGE signaling pathway in diabetic complications (hsa04933), central carbon metabolism in cancer (hsa05230), EGFR tyrosine kinase inhibitor resistance (hsa01521), neurotrophin signaling pathway (hsa04722), fluid shear stress and atherosclerosis (hsa05418), hepatitis B (hsa05161), Kaposi sarcoma-associated herpesvirus infection (hsa05167), FoxO signaling pathway (hsa04068), chemical carcinogenesis-reactive oxygen species (hsa05208), T cell receptor signaling pathway (hsa04660), prolactin signaling pathway (hsa04917), IL-17 signaling pathway (hsa04657), Alzheimer disease (hsa05010), and Ras signaling pathway (hsa04014). These findings highlight the importance of the pathways involved in biological processes, including cancer-related pathways, cardiovascular and metabolic pathways, cellular signaling pathways, infection-related pathways, and neurological pathways. These pathways may play a crucial role in the therapeutic mechanisms of dapagliflozin in the context of CKD.

#### 3.5.2. GO Biological Process Enrichment Analysis

**Figure 4:**
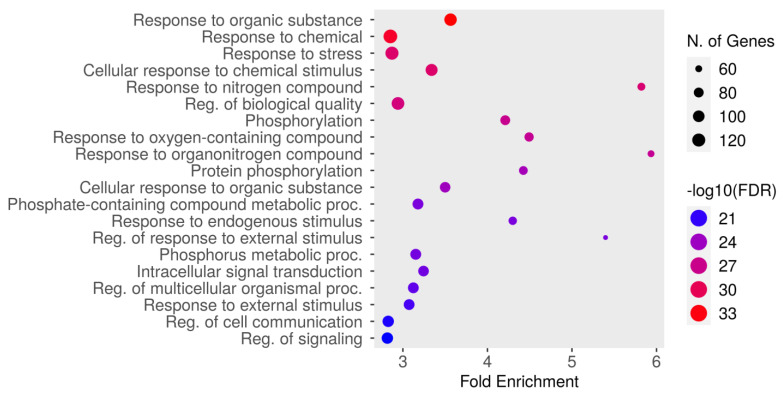
The Gene Ontology (GO) enrichment analysis of biological processes associated with dapagliflozin and chronic kidney disease (CKD). The dot plot displays the top enriched biological processes, ranked by fold enrichment, with the size of each dot representing the number of genes associated with each process. The color gradient indicates the statistical significance (−log10(FDR)), with red representing the highest significance.

The top 20 results from the biological process enrichment analysis in the GO enrichment analysis are presented in Figure 4 and Table 4, associated with the analyzed gene set. The GO enrichment analysis, a powerful tool used for gene function classification and functional distribution prediction, revealed key pathways and processes relevant to the gene set under study. The most enriched pathway was “response to organic substance” (GO:0010033) with an FDR of 4.6987 × 10^−34^ and a fold enrichment of 3.56, followed by “response to chemical” (GO:0042221; FDR = 9.3426 × 10^−33^, fold enrichment = 2.85), and “response to stress” (GO:0006950; FDR = 1.6161Í10-29, fold enrichment = 2.87). Other significantly enriched pathways included cellular response to chemical stimulus (GO:0070887), response to nitrogen compound (GO:1901698), and regulation of biological quality (GO:0065008). Additional notable pathways included phosphorylation (GO:0016310), response to oxygen-containing compound (GO:1901700), and response to organonitrogen compound (GO:0010243). Metabolic and signaling processes, such as protein phosphorylation (GO:0006468), cellular response to organic substance (GO:0071310), and phosphate-containing compound metabolic process (GO:0006796), were also enriched. Furthermore, pathways like response to endogenous stimulus (GO:0009719), regulation of response to external stimulus (GO:0032101), and phosphorus metabolic process (GO:0006793) demonstrated significant enrichment. Other important pathways included intracellular signal transduction (GO:0035556), regulation of multicellular organismal process (GO:0051239), and response to external stimulus (GO:0009605). The pathways “regulation of cell communication” (GO:0010646) and “regulation of signaling” (GO:0023051) were also enriched. These findings highlight the diverse biological processes potentially involved in the therapeutic mechanisms of dapagliflozin, including responses to chemical and environmental stimuli, metabolic regulation, and intracellular signaling.

#### 3.5.3. GO Molecular Function Enrichment Analysis

**Figure 5:**
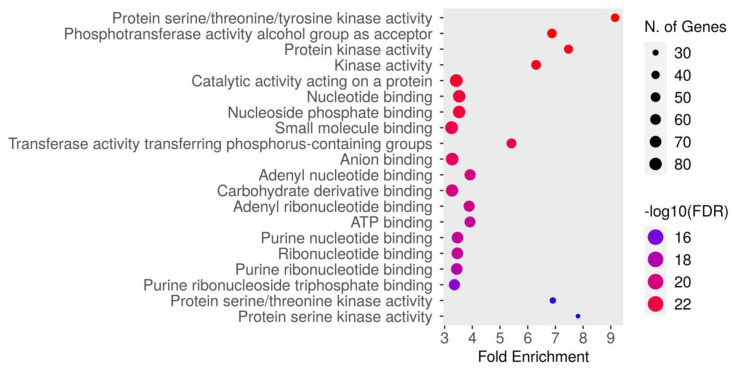
The Gene Ontology (GO) molecular function enrichment analysis of intersecting targets of dapagliflozin and chronic kidney disease (CKD). The top enriched molecular functions are depicted, ranked by fold enrichment and statistical significance (−log10[FDR]). The size of the dots corresponds to the number of genes associated with each pathway, while the color represents the statistical significance, with red indicating higher −log10(FDR) values.

The molecular function enrichment analysis of dapagliflozin revealed significant enrichment of several key activities. As shown in Figure 5 and Table 5, the most enriched function was protein serine/threonine/tyrosine kinase activity (GO:0004712) with an FDR of 1.5084 × 10^−24^. This was followed by the phosphotransferase activity alcohol group as acceptor (GO:0016773), protein kinase activity (GO:0004672), kinase activity (GO:0016301), catalytic activity acting on a protein (GO:0140096), nucleotide binding (GO:0000166), nucleoside phosphate binding (GO:1901265), small molecule binding (GO:0036094), transferase activity transferring phosphorus-containing groups (GO:0016772), anion binding (GO:0043168), adenyl nucleotide binding (GO:0030554), carbohydrate derivative binding (GO:0097367), adenyl ribonucleotide binding (GO:0032559), ATP binding (GO:0005524), purine nucleotide binding (GO:0017076), ribonucleotide binding (GO:0032553), purine ribonucleotide binding (GO:0032555), purine ribonucleoside triphosphate binding (GO:0035639), protein serine/threonine kinase activity (GO:0004674), and protein serine kinase activity (GO:0106310). These findings emphasize the critical molecular functions, particularly kinase activity and nucleotide binding, which may contribute to the therapeutic mechanisms of dapagliflozin in CKD.

#### 3.5.4. GO Cellular Component Enrichment Analysis

**Figure 6:**
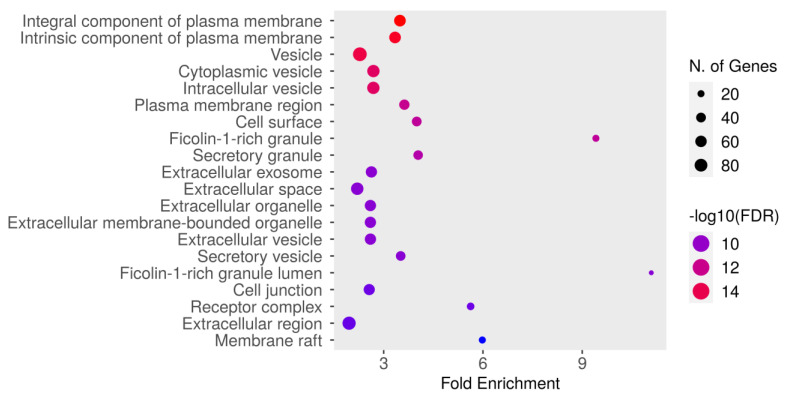
The Gene Ontology (GO) cellular component enrichment analysis of intersecting targets of dapagliflozin and chronic kidney disease (CKD). The size of the dots represents the number of genes associated with each cellular component, and the color indicates the level of statistical significance, with red representing the most significant pathways.

The GO cellular component enrichment analysis for dapagliflozin and chronic kidney disease (CKD) identified key cellular structures and regions significantly associated with the intersecting targets, ranked by FDR values and fold enrichment, as shown in Figure 6 and Table 6. The most enriched component was the integral component of plasma membrane (GO:0005887) with an FDR of 1.8106 × 10^−16^ and a fold enrichment of 3.491. This was closely followed by the intrinsic component of plasma membrane (GO:0031226) and vesicle (GO:0031982). Other significantly enriched components included cytoplasmic vesicle (GO:0031410), intracellular vesicle (GO:0097708), and plasma membrane region (GO:0098590). Enrichment was also observed in cell surface (GO:0009986) and ficolin-1-rich granule (GO:0101002). Further enriched components included secretory granule (GO:0030141) and extracellular exosome (GO:0070062). The analysis also identified extracellular space (GO:0005615), extracellular organelle (GO:0043230), and extracellular vesicle (GO:1903561). Other notable components included secretory vesicle (GO:0099503), ficolin-1-rich granule lumen (GO:1904813), and cell junction (GO:0030054). Additionally, receptor complex (GO:0043235), extracellular region (GO:0005576), and membrane raft (GO:0045121) were significantly enriched. These results highlight the diverse cellular components potentially involved in the mechanisms of dapagliflozin in CKD, with a focus on plasma membrane-related structures, vesicles, and extracellular components critical for cellular communication and signaling.

### 3.6. Molecular Docking Verification

**Figure 7:**
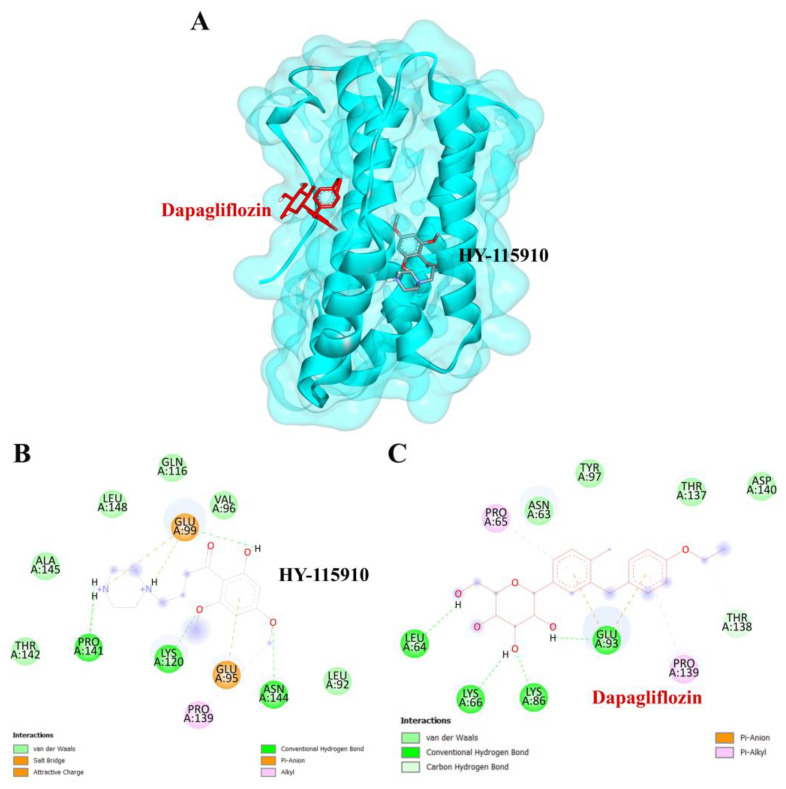
Visualization of the protein–ligand docking interactions. (**A**) Three-dimensional view of dapagliflozin (red) and HY-115910 (black) docked with IL-6. (**B**) Two-dimensional schematic of the docking interaction between HY-115910 (positive control) and IL-6. (**C**) Two-dimensional schematic of the docking interaction between dapagliflozin and IL-6.

**Figure 8:**
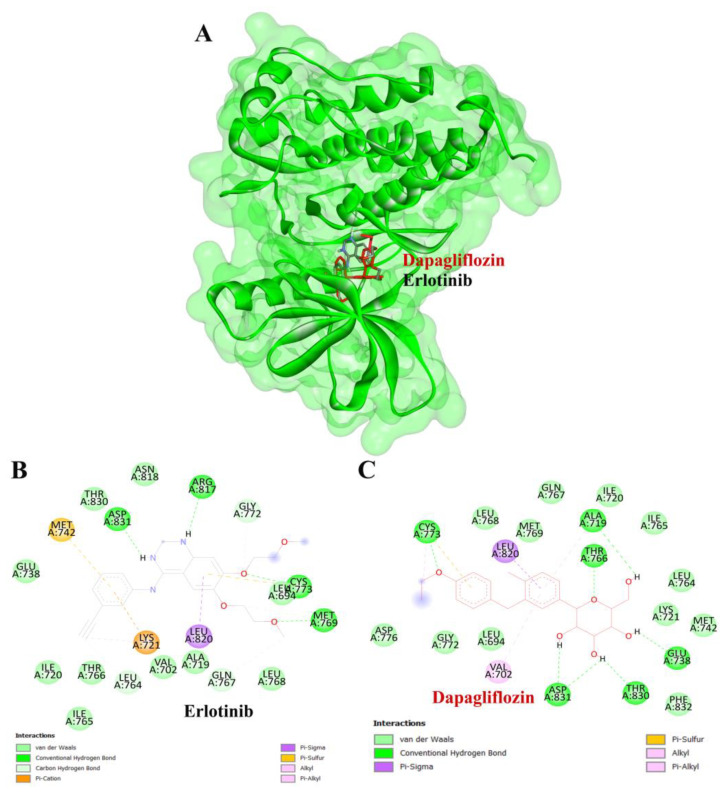
Visualization of the protein–ligand docking interactions. (**A**) Three-dimensional view of dapagliflozin (red) and erlotinib (black) docked with EGFR. (**B**) Two-dimensional schematic of the docking interaction between erlotinib (positive control) and EGFR. (**C**) Two-dimensional schematic of the docking interaction between dapagliflozin and EGFR.

**Figure 9:**
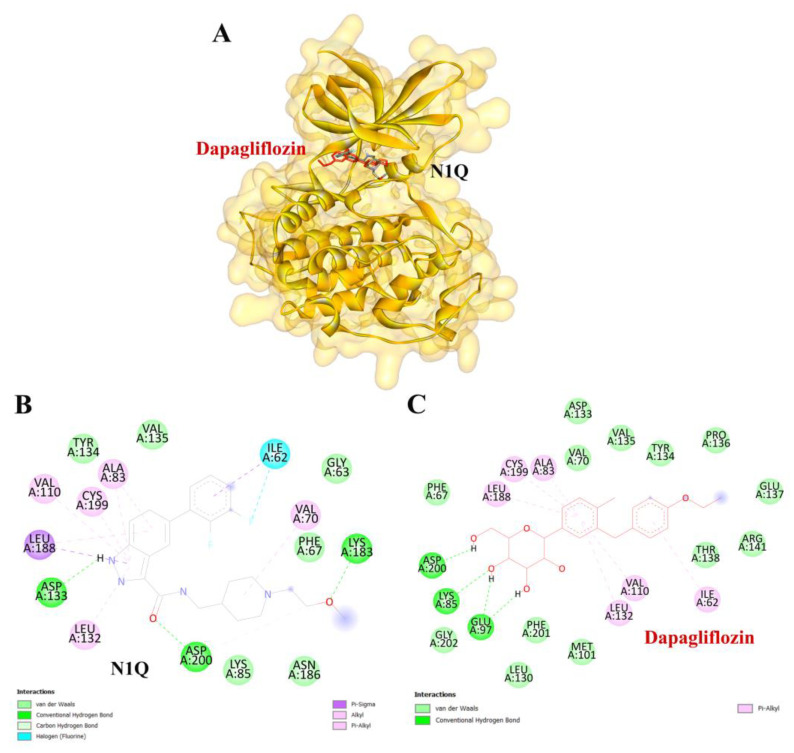
Visualization of the protein–ligand docking interactions. (**A**) A 3D structure showing the docking of dapagliflozin (in red) and N1Q (in black) with the GSK3β protein. (**B**) A 2D schematic of N1Q (positive control) binding with GSK3β. (**C**) A 2D schematic of the docking interaction between dapagliflozin and GSK3β.

To validate the credibility of drug–target interactions, molecular docking analysis was specifically conducted on the 10 hub proteins selected as targets. In this study, the stability and inhibitory potency of the ligand–receptor binding were assessed based on the binding energies between the ligand and protein. A binding energy below −8.0 kcal/mol or a docking score better than that of the positive control served as the criterion or cutoff, indicating a robust conformation of ligand binding to the receptor. The results of molecular docking for dapagliflozin are summarized in Table 7. Dapagliflozin exhibited significant inhibition against EGFR and GSK3β proteins, with docking scores of −8.42 and −7.70 kcal/mol, respectively. Moreover, dapagliflozin demonstrated a notable binding affinity to IL-6, with a binding energy better than that of HY-115910 (positive control). However, dapagliflozin bound to IL-6 at a different binding site compared to HY-115910 (Figure 7A,B). Additionally, dapagliflozin formed five hydrogen bonds with LEU64, LYS66, LYS86, GLU93, and THR138 of IL-6, along with hydrophobic interactions involving PRO65 and PRO139, as depicted in Figure 7C. As shown in Figure 8A, dapagliflozin also exhibited strong binding affinity against EGFR by binding at a site similar to that of erlotinib. Dapagliflozin formed seven hydrogen bonds with CYS773, THR766, ALA719, GLU738, THR830, and two with ASP831 (Figure 8C). Furthermore, dapagliflozin engaged in several hydrophobic interactions with CYS773, LEU820, ALA719, and VAL702 (Figure 8C). Notably, dapagliflozin demonstrated a significant binding affinity to GSK3β, with a binding energy of −7.70 kcal/mol, which was better than that of the positive control N1Q. Dapagliflozin formed four hydrogen bonds with ASP200, LYS85, and two with GLU97 in its interaction with GSK3β, binding at the same site as the positive control drug (Figure 9A–C). These findings highlight the bioactive potential of dapagliflozin, which exhibited notable binding energy and interactions with the top three key hub targets, indicating its significance in modulating these crucial molecular targets.

## 4. Discussion

This study demonstrates the therapeutic potential of dapagliflozin in CKD by identifying 208 shared molecular targets through network pharmacology approaches. These targets, derived from a combination of dapagliflozin-related and CKD-related gene datasets, highlight the drug’s ability to modulate key biological processes implicated in CKD. The analysis of the PPI network identified ten hub proteins, including GAPDH, IL-6, SRC, EGFR, HSP90AA1, NFKB1, CASP3, HSP90AB1, MAPK3, and GSK3β, which are central nodes in the network and may serve as pivotal mediators of dapagliflozin’s therapeutic effects [16].

The ADMET analysis affirmed dapagliflozin’s favorable pharmacokinetic and toxicity profiles, establishing it as a promising oral therapeutic for CKD. The drug demonstrated high gastrointestinal absorption, low toxicity risks, and no violations of Lipinski’s rule of five, ensuring good oral bioavailability [31]. Additionally, its low cardiac and hepatotoxicity profiles further reinforce its suitability for long-term use. The GO enrichment analysis provided valuable insights into biological processes, molecular functions, and cellular components related to dapagliflozin’s targets. Enriched biological processes included responses to chemical stimuli, signaling regulation, and metabolic processes, underscoring its role in mitigating inflammation and oxidative stress. Molecular function analysis emphasized kinase activity, nucleotide binding, and catalytic activity, which are essential for intracellular signaling and cellular regulation. Cellular component enrichment highlighted the significance of plasma membrane structures, vesicles, and extracellular regions as mediators of the drug’s effects [32]. The KEGG pathway analysis identified critical pathways, such as the PI3K-Akt signaling pathway, MAPK signaling pathway, and AGE-RAGE signaling in diabetic complications, which are closely associated with CKD pathophysiology [33,34,35]. The PI3K-Akt pathway is involved in renal cell survival, proliferation, and immune responses, and its dysregulation contributes to renal fibrosis, chronic inflammation, and oxidative damage. Excessive activation of PI3K-Akt promotes epithelial-to-mesenchymal transition (EMT) and mitochondrial dysfunction, exacerbating renal injury [33]. Conversely, the inhibition of this pathway has been shown to mitigate renal fibrosis and inflammation. Similarly, the MAPK pathway is implicated in CKD progression by inducing pro-inflammatory cytokines and fibrotic mediators, leading to tubulointerstitial fibrosis and glomerulosclerosis [36]. MAPK activation triggers oxidative stress and apoptosis in renal tubular cells, further contributing to CKD pathogenesis. The AGE-RAGE pathway, particularly relevant in diabetic nephropathy, accelerates CKD progression through the accumulation of advanced glycation end-products (AGEs), which activate NF-κB signaling, increase oxidative stress, and induce endothelial dysfunction and podocyte injury, resulting in inflammation and fibrosis, promoting proteinuria and renal function decline [35]. These pathways, involved in cellular proliferation, oxidative stress, inflammation, and metabolic regulation, provide mechanistic insights into dapagliflozin’s diverse effects.

While direct evidence of dapagliflozin’s modulation of the PI3K-Akt, MAPK, and AGE-RAGE pathways specifically in CKD models is limited, several studies suggest its influence on these pathways in related contexts. A recent in vitro study demonstrated that dapagliflozin attenuates isoproterenol-induced hypertrophy in cardiomyocytes by activating the AKT pathway, leading to reduced oxidative stress and inflammation. In aortic endothelial cells, dapagliflozin restored AKT and PI3K expression, enhanced MAPK activation, and downregulated inflammatory cytokines, indicating its potential to preserve vascular function and improve endothelial health. Additionally, dapagliflozin upregulated NRF2 expression, positively influencing β-cell function and stress response [37]. Regarding the AGE-RAGE pathway, the activation of RAGE is increased in CKD and contributes to cellular dysfunction, tissue injury, fibrosis, and inflammation. While direct modulation of the AGE-RAGE pathway by dapagliflozin in CKD models has not been conclusively demonstrated, the drug’s anti-inflammatory and antioxidative properties may indirectly influence this pathway, potentially mitigating RAGE-mediated effects [37]. Regarding the AGE-RAGE pathway, the activation of RAGE is increased in CKD and contributes to cellular dysfunction, tissue injury, fibrosis, and inflammation. While direct modulation of the AGE-RAGE pathway by dapagliflozin in CKD models has not been conclusively demonstrated, the drug’s anti-inflammatory and antioxidative properties may indirectly influence this pathway, potentially mitigating RAGE-mediated effects [38].

Findings from the DAPA-CKD trial provide strong clinical evidence of dapagliflozin’s renoprotective effects beyond glucose lowering. In this trial, dapagliflozin significantly reduced CKD progression by 39% and all-cause mortality by 31%, which are benefits observed regardless of diabetes status. These findings suggest that dapagliflozin may indirectly influence inflammation, oxidative stress, and fibrotic pathways, which are central to CKD progression [10]. Similarly, the DAPA-HF trial showed a 26% reduction in cardiovascular death or heart failure hospitalization, further supporting its disease-modulating effects [39]. Other pathways related to lipid metabolism, atherosclerosis, and cardiovascular processes further support the potential of dapagliflozin to manage CKD complications [40].

Interestingly, our analysis revealed a potential connection between prostate cancer, hepatitis B, and CKD progression. We hypothesize that this association may be attributed to shared pathophysiological mechanisms, including inflammation, immune activation, and cellular proliferation. Prostate cancer has been linked to a higher incidence of acute kidney injury (AKI) and CKD, with studies reporting a 1.47-fold increased risk of AKI in prostate cancer patients compared to those without cancer. Additionally, androgen deprivation therapy, a common treatment for prostate cancer, has been associated with renal complications, further supporting a possible connection between prostate cancer-related pathways and CKD progression [41]. Moreover, chronic hepatitis B virus (HBV) infection is a recognized risk factor for CKD development, with HBV-infected individuals exhibiting a higher prevalence of CKD and glomerular diseases, such as membranous nephropathy and polyarteritis nodosa, contributing to renal injury and dysfunction [42]. The identification of prostate cancer and hepatitis B pathways in our KEGG analysis suggests that these conditions may have a broader impact on renal pathology.

In a previous study, network pharmacology was utilized to examine the therapeutic roles of canagliflozin and dapagliflozin in atherosclerosis, revealing that these drugs act by targeting key molecules such as Akt1, MAPK1, MAPK14, SRC, and EGFR [43]. Building on this, our recent investigation employed network pharmacology and molecular docking to explore the therapeutic potential of dapagliflozin in chronic kidney disease (CKD). By integrating data from multiple databases, we identified 208 CKD-related targets for dapagliflozin, focusing specifically on the ten most interconnected proteins, which are integral to the biological mechanisms underlying CKD pathogenesis. These findings underscore dapagliflozin’s ability to modulate diverse biological pathways and functions relevant to CKD progression. Clinical evidence further supports dapagliflozin’s efficacy, demonstrating a significant reduction in the risk of a 50% or greater decline in the estimated glomerular filtration rate (eGFR), progression to end-stage kidney disease, or death from renal or cardiovascular causes, compared to the placebo, in CKD patients regardless of diabetes status [10]. Additionally, in patients with stage 4 CKD and albuminuria, dapagliflozin consistently mitigated major kidney and cardiovascular risks and slowed eGFR decline without increasing adverse outcomes, aligning with broader trial findings [44]. The results underscore dapagliflozin’s significance as a therapeutic option for effectively managing CKD in a wide range of patient populations.

To complement our network pharmacology analysis, molecular docking revealed significant binding affinities between dapagliflozin and key proteins. The analysis confirmed dapagliflozin’s high binding affinity for critical targets, including EGFR, GSK3β, and IL-6. Notably, dapagliflozin exhibited a binding energy of −8.42 kcal/mol with EGFR, surpassing that of erlotinib. This interaction was facilitated by hydrogen bonds and hydrophobic interactions, indicating a stable ligand-receptor conformation (Table 2). The EGFR pathway plays a vital role in kidney development, tissue repair, and electrolyte balance. Dysregulation of this pathway has been extensively linked to the onset and progression of chronic kidney diseases, such as diabetic nephropathy, chronic allograft nephropathy, and polycystic kidney disease. This is attributed to its involvement in promoting renal cell proliferation, fibrosis, and inflammation. Experimental evidence strongly supports that aberrant EGFR signaling contributes to many forms of chronic kidney disease. Furthermore, abnormal EGFR activation has been implicated in mediating progressive kidney injury, particularly in diabetic kidney disease [45]. Inhibiting EGFR expression or activity has been shown to slow the progression of diabetic kidney injury, suggesting that directly targeting EGFR or its associated signaling pathways may be an effective strategy for preventing progressive kidney damage caused by diabetes [46].

Similarly, dapagliflozin demonstrated a high level of inhibition toward glycogen synthase kinase 3 beta (GSK3β), with a docking score of −7.70 kcal/mol, outperforming the positive control, N1Q. The strong binding affinities with both EGFR and GSK3β underscore dapagliflozin’s potential as a multi-target therapeutic agent in CKD. GSK3β contributes to mitochondrial damage and apoptosis by directly phosphorylating and activating pro-apoptotic proteins Bax and Caspase-3 [47]. Additionally, GSK3β activates the p65 subunit of NF-κB, thereby amplifying inflammatory processes [48]. It also inhibits Nrf2, a key antioxidant defender, by phosphorylating it and promoting its nuclear exclusion. This suppression of Nrf2 leads to oxidative stress in the kidney and impedes the transition from acute kidney injury (AKI) to CKD [49]. Furthermore, GSK3β-mediated Keap1-independent regulation of the Nrf2 antioxidant response acts as a molecular rheostat in the AKI-to-CKD transition [50]. These processes slow tubular recovery and contribute to the progression from AKI to CKD [51].

Dapagliflozin also exhibited significant binding affinity to IL-6, surpassing the binding energy of the positive control, HY-115910. Circulating interleukin-6 (IL-6) levels are often elevated in type 2 diabetes mellitus (T2DM) [52]. Increasing evidence highlights the critical role of IL-6 in the pathophysiology of cardiovascular and renal dysfunction [53], with IL-6 being recognized as a major contributor to kidney diseases [54]. Notably, dapagliflozin has been shown to reduce IL-6 levels more effectively than other medications. The anti-inflammatory properties of SGLT2 inhibitors, including dapagliflozin, support their broader use in managing diabetic complications related to kidney inflammation [55,56,57]. Although EGFR activation contributes to tubulointerstitial fibrosis and renal dysfunction, direct evidence of dapagliflozin modulating this pathway in CKD is currently lacking. However, its anti-inflammatory and antifibrotic properties suggest potential indirect effects. Similarly, IL-6, a key pro-inflammatory cytokine in CKD, drives systemic inflammation and endothelial dysfunction. Clinical studies indicate that dapagliflozin reduces IL-6 levels in patients with CKD and diabetes, highlighting a direct anti-inflammatory effect. This aligns with dapagliflozin’s broader anti-inflammatory profile, reinforcing the relevance of IL-6 in CKD pathophysiology [58]. This study integrates network pharmacology and molecular docking to offer a comprehensive understanding of dapagliflozin’s mechanisms of action. It underscores the drug’s potential to function beyond its primary role as an SGLT2 inhibitor, providing a promising approach for CKD treatment through multi-target modulation.

The findings from this study also provide a strong foundation for future experimental research and clinical applications in CKD treatment. The identification of 208 overlapping molecular targets and key signaling pathways, including PI3K-Akt, MAPK, and AGE-RAGE, suggests potential therapeutic mechanisms for dapagliflozin. Future in vitro and in vivo studies should focus on validating these pathways’ roles in CKD progression and assessing dapagliflozin’s efficacy in modulating them. Additionally, given its ability to target multiple aspects of CKD pathophysiology—such as inflammation, oxidative stress, and metabolic dysregulation—dapagliflozin could be explored as part of combination therapies to enhance renoprotective effects. Investigating its interactions with key proteins such as EGFR, IL-6, and GSK3β may offer new insights into optimizing treatment strategies.

Furthermore, personalized medicine approaches should be considered to identify patient-specific factors that influence drug response. Biomarker-driven studies may help stratify patients based on CKD progression risk and predict responsiveness to dapagliflozin. While clinical trials have demonstrated its efficacy in reducing renal injury markers and slowing eGFR decline, additional studies are needed to evaluate its benefits in broader CKD populations, including non-diabetic CKD, polycystic kidney disease, and transplant recipients. Long-term trials should assess its role in delaying dialysis initiation and reducing cardiovascular events. Additionally, novel drug formulations, such as nanotechnology-based delivery systems, could be explored to improve dapagliflozin’s bioavailability and target specificity while minimizing systemic side effects.

## 5. Conclusions

This study demonstrates the potential of dapagliflozin as a therapeutic agent for chronic kidney disease (CKD) by identifying 208 overlapping targets and elucidating its multi-target mechanisms through network pharmacology and molecular docking. Key pathways, including PI3K-Akt, MAPK, and AGE-RAGE signaling, were identified as critical to CKD pathophysiology, with hub proteins such as EGFR, IL-6, and GSK3β validated for their strong binding affinity to dapagliflozin. These findings highlight dapagliflozin’s ability to modulate inflammation, oxidative stress, and metabolic dysregulation, suggesting that its therapeutic utility extends beyond its established role as an SGLT2 inhibitor. This study emphasizes the value of computational approaches in drug discovery and provides a foundation for the further experimental validation and clinical application of dapagliflozin in CKD management.

## Figures and Tables

**Table 1:** Details of the protein targets in the PDB database and the grid docking parameters in molecular docking.

Targets	PDB ID	Method	Resolution (Å)	R-Value Free	R-Value Work	Spacing (Å)	Center Grid Box		
							X Center	Y Center	Z Center
GAPDH	6M61	X-ray diffraction	1.82	0.228	0.192	0.375	−15.666	6.041	−22.87
IL6	1ALU	X-ray diffraction	1.90	0.277	0.213	0.375	2.836	−19.593	9.095
SRC	1Y57	X-ray diffraction	1.91	0.213	0.188	0.375	12.093	34.632	39.141
EGFR	1M17	X-ray diffraction	2.60	0.295	0.251	0.375	24.838	0.016	53.366
HSP90AA1	4AWQ	X-ray diffraction	1.60	0.262	0.227	0.375	−2.926	4.130	−4.899
NFKB	3GUT	X-ray diffraction	3.59	0.301	0.245	0.375	30.231	−27.423	64.005
CASP3	3KJF	X-ray diffraction	2.00	0.206	0.181	0.375	16.346	−5.814	3.906
HSP0AB1	5UCJ	X-ray diffraction	1.69	0.197	0.174	0.375	−24.841	99.831	0.583
MAPK3	4QTB	X-ray diffraction	1.40	0.175	0.147	0.375	33.748	54.397	49.771
GSK3B	6TCU	X-ray diffraction	2.14	0.240	0.202	0.375	−14.502	−14.985	−0.926

**Table 2:** The 208 overlapping targets of dapagliflozin and CKD.

Target	Common Name
Multidrug resistance-associated protein 1	ABCC1
Tyrosine-protein kinase ABL1	ABL1
Acetylcholinesterase	ACHE
Activin receptor type-1-like	ACVRL1
Disintegrin and metalloproteinase domain-containing protein 10	ADAM10
Adenosine kinase	ADK
Adenosine receptor A1	ADORA1
Adenosine receptor A2a	ADORA2A
Adenosine receptor A2b	ADORA2B
Adenosine receptor A3	ADORA3
Adenosylhomocysteinase	AHCY
Alkaline phosphatase, tissue-nonspecific isozyme	ALPL
Amine oxidase [copper containing] 3	AOC3
DNA repair nuclease/redox regulator APEX1	APEX1
Cysteine protease ATG4B	ATG4B
Aurora kinase B	AURKB
RecQ-like DNA helicase BLM	BLM
Serine/threonine-protein kinase B-raf	BRAF
C5a anaphylatoxin chemotactic receptor 1	C5AR1
Carbonic anhydrase 14	CA14
Voltage-gated N-type calcium channel alpha-1B subunit	CACNA1B
Voltage-gated T-type calcium channel alpha-1H subunit	CACNA1H
CaM-kinase kinase beta	CAMKK2
Calpain 1	CAPN1
Histone-arginine methyltransferase CARM1	CARM1
Caspase-3	CASP3
G2/mitotic-specific cyclin-B1	CCNB1
G1/S-specific cyclin-E1	CCNE1
Cyclin H	CCNH
Cyclin T1	CCNT1
Cell division cycle 7-related protein kinase	CDC7
Cyclin-dependent kinase 1	CDK1
Cyclin-dependent kinase 2	CDK2
Cyclin-dependent kinase 7	CDK7
Cyclin-dependent kinase 9	CDK9
Cystic fibrosis transmembrane conductance regulator	CFTR
Serine/threonine-protein kinase Chk1	CHEK1
Conserved helix-loop-helix ubiquitous kinase	CHUK
Cannabinoid receptor 1	CNR1
Cannabinoid receptor 2	CNR2
Casein kinase I isoform alpha	CSNK1A1
Casein kinase II subunit alpha	CSNK2A1
Cathepsin D	CTSD
Cathepsin L	CTSL
C-X-C chemokine receptor type 2	CXCR2
C-X-C chemokine receptor type 3	CXCR3
Cysteinyl leukotriene receptor 2	CYSLTR2
Dihydroorotate dehydrogenase	DHODH
DNA (cytosine-5)-methyltransferase 1	DNMT1
Dipeptidyl peptidase 2	DPP7
Dipeptidyl peptidase 9	DPP9
Dual-specificity tyrosine-phosphorylation-regulated kinase 1A	DYRK1A
Epidermal growth factor receptor	EGFR
Ephrin type-A receptor 5	EPHA5
Endoplasmic reticulum aminopeptidase 1	ERAP1
Coagulation factor XIII A chain	F13A1
Coagulation factor VII/tissue factor	F3
Fatty-acid amide hydrolase 1	FAAH
Fatty acid binding protein, adipocyte	FABP4
Squalene synthetase	FDFT1
Free fatty acid receptor 4	FFAR4
Fibroblast growth factor 1	FGF1
Fibroblast growth factor 2	FGF2
Fibroblast growth factor receptor 1	FGFR1
Tissue alpha-L-fucosidase	FUCA1
Lysosomal alpha-glucosidase	GAA
Gamma-aminobutyric acid receptor subunit alpha-1	GABRA1
Gamma-aminobutyric acid receptor subunit alpha-5	GABRA5
Cyclin-G-associated kinase	GAK
Glyceraldehyde-3-phosphate dehydrogenase	GAPDH
Lysosomal acid glucosylceramidase	GBA1
Non-lysosomal glucosylceramidase	GBA2
Geranylgeranyl pyrophosphate synthetase	GGPS1
Beta-galactosidase	GLB1
Glycine receptor subunit alpha-1	GLRA1
Glutaminase kidney isoform, mitochondrial	GLS
G-protein coupled bile acid receptor 1	GPBAR1
Uracil nucleotide/cysteinyl leukotriene receptor	GPR17
G-protein coupled receptor 35	GPR35
Growth factor receptor-bound protein 2	GRB2
Glutamate receptor 2	GRIA2
Glutamate receptor ionotropic, kainate 1	GRIK1
G protein-regulated inducer of neurite outgrowth 1	GRIN1
Metabotropic glutamate receptor 4	GRM4
Glycogen synthase kinase-3 beta	GSK3B
Glutathione S-transferase Mu 1	GSTM1
Glutathione S-transferase P	GSTP1
Beta-glucuronidase	GUSB
Glycogen [starch] synthase, muscle	GYS1
Histone deacetylase 5	HDAC5
Histone deacetylase 7	HDAC7
Hexokinase-1	HK1
Hexokinase-2	HK2
15-hydroxyprostaglandin dehydrogenase [NAD+]	HPGD
Hypoxanthine-guanine phosphoribosyltransferase	HPRT1
GTPase HRas	HRAS
Histamine H3 receptor	HRH3
3-hydroxyacyl-CoA dehydrogenase type-2	HSD17B10
Heat shock protein HSP 90-alpha	HSP90AA1
Heat shock protein HSP 90-beta	HSP90AB1
Endoplasmic reticulum chaperone BiP	HSPA5
Heat shock cognate 71 kDa protein	HSPA8
Intercellular adhesion molecule-1	ICAM1
Interleukin-2	IL2
Interleukin-6	IL6
Interleukin-1 receptor-associated kinase 4	IRAK4
Integrin alpha-L	ITGAL
Integrin beta-1	ITGB1
Integrin beta-2	ITGB2
Tyrosine-protein kinase ITK/TSK	ITK
Tyrosine-protein kinase JAK2	JAK2
Potassium voltage-gated channel subfamily A member 5	KCNA5
Lysine-specific histone demethylase 1A	KDM1A
Lysine-specific demethylase 4A	KDM4A
Lysine-specific demethylase 4C	KDM4C
Kruppel-like factor 5	KLF5
Kallikrein-1	KLK1
Plasma kallikrein	KLKB1
Tyrosine-protein kinase LCK	LCK
L-lactate dehydrogenase A chain	LDHA
Galectin-1	LGALS1
Galectin-3	LGALS3
Leukotriene A-4 hydrolase	LTA4H
Dual specificity mitogen-activated protein kinase 1	MAP2K1
Mitogen-activated protein kinase 1	MAPK1
Mitogen-activated protein kinase 10	MAPK10
Mitogen-activated protein kinase 11	MAPK11
Mitogen-activated protein kinase 14	MAPK14
Mitogen-activated protein kinase 15	MAPK15
Mitogen-activated protein kinase 3	MAPK3
Mitogen-activated protein kinase 8	MAPK8
Hepatocyte growth factor receptor	MET
Maltase-glucoamylase	MGAM
Macrophage migration inhibitory factor	MIF
Neprilysin	MME
Matrix metalloproteinase 1	MMP1
Matrix metalloproteinase 3	MMP3
NEDD8-activating enzyme E1 regulatory subunit	NAE1
Nuclear factor erythroid 2-related factor 2	NFE2L2
Nuclear factor NF-kappa-B p105 subunit	NFKB1
Tumor necrosis factor receptor superfamily member 16	NGFR
Nitric oxide synthase, inducible	NOS2
NADPH oxidase 1	NOX1
Nuclear receptor subfamily 1 group I member 2	NR1I2
Mineralocorticoid receptor	NR3C2
GTPase NRas	NRAS
5′-nucleotidase	NT5E
High affinity nerve growth factor receptor	NTRK1
NT-3 growth factor receptor	NTRK3
Protein O-GlcNAcase	OGA
P2X purinoceptor 4	P2RX4
P2Y purinoceptor 12	P2RY12
Phosphodiesterase 5A	PDE5A
Phosphodiesterase 11A	PDE11A
Platelet-derived growth factor receptor beta	PDGFRB
6-phosphofructo-2-kinase/fructose-2,6-bisphosphatase 3	PFKFB3
Phosphatidylinositol 4,5-bisphosphate 3-kinase catalytic subunit beta isoform	PIK3CB
Phosphatidylinositol 4,5-bisphosphate 3-kinase catalytic subunit gamma isoform	PIK3CG
Tissue-type plasminogen activator	PLAT
Urokinase-type plasminogen activator	PLAU
Lysosomal Pro-X carboxypeptidase	PRCP
DNA-dependent protein kinase catalytic subunit	PRKDC
Protein-arginine N-methyltransferase 1	PRMT1
Protein arginine N-methyltransferase 7	PRMT7
Serine protease 1	PRSS1
Proteasome subunit beta type-1	PSMB1
Proteasome subunit beta type-2	PSMB2
Prostaglandin E2 receptor EP1 subtype	PTGER1
Prostaglandin G/H synthase 1	PTGS1
Tyrosine-protein phosphatase non-receptor type 1	PTPN1
Tyrosine-protein phosphatase non-receptor type 7	PTPN7
Glycogen phosphorylase, liver form	PYGL
Glycogen phosphorylase, muscle form	PYGM
Nuclear receptor ROR-beta	RORB
Sphingosine 1-phosphate receptor 3	S1PR3
Sphingosine 1-phosphate receptor 4	S1PR4
SUMO-activating enzyme subunit 1	SAE1
Stearoyl-CoA desaturase	SCD
Sodium channel protein type 4 subunit alpha	SCN4A
E-Selectin	SELE
Sucrase-isomaltase	SI
Excitatory amino acid transporter 1	SLC1A3
Sodium/nucleoside cotransporter 2	SLC28A2
Equilibrative nucleoside transporter 1	SLC29A1
Solute carrier family 2, facilitated glucose transporter member 1	SLC2A1
Sodium/glucose cotransporter 1	SLC5A1
Sodium/myo-inositol cotransporter 2	SLC5A11
Sodium/glucose cotransporter 2	SLC5A2
Probable glucose sensor protein SLC5A4	SLC5A4
Sodium-dependent noradrenaline transporter	SLC6A2
Sodium-dependent serotonin transporter	SLC6A4
Sodium- and chloride-dependent glycine transporter 2	SLC6A5
Proto-oncogene tyrosine-protein kinase Src	SRC
Stimulator of interferon genes protein	STING1
Tyrosine-protein kinase SYK	SYK
Substance-K receptor	TACR2
Tyrosyl-DNA phosphodiesterase 1	TDP1
Tissue factor pathway inhibitor	TFPI
Thyroid hormone receptor alpha	THRA
Thymidine kinase 2, mitochondrial	TK2
Transmembrane protease serine 6	TMPRSS6
DNA topoisomerase 2-alpha	TOP2A
Transcription intermediary factor 1-alpha	TRIM24
Tyrosinase	TYR
Tyrosine-protein kinase receptor TYRO3	TYRO3
Ubiquitin-like modifier-activating enzyme 7	UBA7
Vascular cell adhesion protein 1	VCAM1
Vascular endothelial growth factor A, long form	VEGFA

**Table 3:** The Kyoto Encyclopedia of Genes and Genomes (KEGG) pathway enrichment analysis of 208 overlapping targets between dapagliflozin and chronic kidney disease.

Pathway	Description	Number of Genes	Pathway Genes	Fold Enrichment	Enrichment FDR
hsa05215	Prostate cancer	22	97	23.81	5.9925 × 10^−22^
hsa05417	Lipid and atherosclerosis	28	214	13.73	1.2173 × 10^−21^
hsa05200	Pathways in cancer	38	530	7.53	1.7505 × 10^−20^
hsa04015	Rap1 signaling pathway	25	210	12.50	1.8249 × 10^−18^
hsa04010	MAPK signaling pathway	28	294	10.00	3.1128 × 10^−18^
hsa04151	PI3K-Akt signaling pathway	30	354	8.89	3.1609 × 10^−18^
hsa04933	AGE-RAGE signaling pathway in diabetic complications	19	100	19.94	4.1871 × 10^−18^
hsa05230	Central carbon metabolism in cancer	17	70	25.49	4.1871 × 10^−18^
hsa01521	EGFR tyrosine kinase inhibitor resistance	17	79	22.59	3.4162 × 10^−17^
hsa04722	Neurotrophin signaling pathway	19	119	16.76	9.6469 × 10^−17^
hsa05418	Hepatitis B	20	138	15.21	9.6469 × 10^−17^
hsa05161	Kaposi sarcoma-associated herpesvirus infection	21	162	13.61	1.2309 × 10^−16^
hsa05167	Chemical carcinogenesis-reactive oxygen species	22	194	11.90	3.3219 × 10^−16^
hsa04068	FoxO signaling pathway	19	131	15.22	4.5985 × 10^−16^
hsa05208	Chemical carcinogenesis-reactive oxygen species	23	223	10.83	4.5985 × 10^−16^
hsa04660	T cell receptor signaling pathway	17	103	17.32	2.2045 × 10^−15^
hsa04917	Prolactin signaling pathway	15	70	22.49	2.2045 × 10^−15^
hsa04657	L-17 signaling pathway	16	93	18.06	8.1213 × 10^−15^
hsa05010	Alzheimer disease	27	384	7.38	8.8190 × 10^−15^
hsa04014	Ras signaling pathway	22	235	9.83	1.3050 × 10^−14^

**Table 4:** The GO biological process enrichment analysis of targets shared by dapagliflozin and chronic kidney disease.

GO	Description	Number of Genes	Pathway Genes	Fold Enrichment	Enrichment FDR
GO:0010033	Response to organic substance	111	3269	3.56	4.6987 × 10^−34^
GO:0042221	Response to chemical	131	4821	2.85	9.3426 × 10^−33^
GO:0006950	Response to stress	121	4424	2.87	1.6161 × 10^−29^
GO:0070887	Cellular response to chemical stimulus	105	3300	3.34	1.6161 × 10^−29^
GO:1901698	Response to nitrogen compound	65	1172	5.82	3.8489 × 10^−29^
GO:0065008	GO:0065008 regulation of biological quality	115	4103	2.94	1.5913 × 10^−28^
GO:0016310	Phosphorylation	80	1994	4.21	1.7416 × 10^−27^
GO:1901700	Response to oxygen-containing compound	75	1752	4.49	3.0411 × 10^−27^
GO:0010243	Response to organonitrogen compound	60	1061	5.94	3.0857 × 10^−27^
GO:0006468	Protein phosphorylation	71	1684	4.43	3.5421 × 10^−25^
GO:0071310	Cellular response to organic substance	87	2609	3.50	7.9542 × 10^−25^
GO:0006796	Phosphate-containing compound metabolic process	91	3005	3.18	2.8057 × 10^−23^
GO:0009719	Response to endogenous stimulus	68	1660	4.30	2.8057 × 10^−23^
GO:0032101	Reg. of response to external stimulus	56	1089	5.40	2.8057 × 10^−23^
GO:0006793	Phosphorus metabolic process	91	3030	3.15	4.5212 × 10^−23^
GO:0035556	Intracellular signal transduction	88	2847	3.24	5.4800 × 10^−23^
GO:0051239	Regulation of multicellular organismal process	90	3024	3.12	1.6576 × 10^−22^
GO:0009605	Response to external stimulus	90	3073	3.07	4.9737 × 10^−22^
GO:0010646	Regulation of cell communication	97	3602	2.83	1.4809 × 10^−21^
GO:0023051	Regulation of signaling	97	3615	2.82	1.8495 × 10^−21^

**Table 5:** The GO molecular function enrichment analysis of intersecting targets shared by dapagliflozin and chronic kidney disease.

GO	Description	Number of Genes	Pathway Genes	Fold Enrichment	Enrichment FDR
GO:0004712	Protein serine/threonine/tyrosine kinase activity	41	470	9.16	1.5084 × 10^−24^
GO:0016773	Phosphotransferase activity alcohol group as acceptor	49	748	6.88	1.5688 × 10^−24^
GO:0004672	Protein kinase activity	45	632	7.47	4.2997 × 10^−24^
GO:0016301	Kinase activity	51	849	6.30	4.2997 × 10^−24^
GO:0140096	Catalytic activity acting on a protein	84	2577	3.42	1.7598 × 10^−23^
GO:0000166	Nucleotide binding	80	2381	3.53	4.4649 × 10^−23^
GO:1901265	Nucleoside phosphate binding	80	2382	3.53	4.4649 × 10^−23^
GO:0036094	Small molecule binding	85	2743	3.25	1.5947 × 10^−22^
GO:0016772	Transferase activity transferring phosphorus-containing groups	52	1008	5.41	5.0793 × 10^−22^
GO:0043168	Anion binding	82	2630	3.27	8.4681 × 10^−22^
GO:0030554	Adenyl nucleotide binding	65	1741	3.92	1.4539 × 10^−20^
GO:0097367	Carbohydrate derivative binding	78	2505	3.27	1.5727 × 10^−22^
GO:0032559	Adenyl ribonucleotide binding	64	1729	3.89	4.5342 × 10^−22^
GO:0005524	ATP binding	62	1662	3.92	1.5083 × 10^−19^
GO:0017076	Purine nucleotide binding	70	2120	3.47	1.6300 × 10^−19^
GO:0032553	Ribonucleotide binding	70	2123	3.46	1.6547 × 10^−19^
GO:0032555	Purine ribonucleotide binding	69	2106	3.44	4.7366 × 10^−19^
GO:0035639	Purine ribonucleoside triphosphate binding	65	2034	3.35	3.1939 × 10^−17^
GO:0004674	Protein serine/threonine kinase activity	31	471	6.91	1.0688 × 10^−15^
GO:0106310	Protein serine kinase activity	28	376	7.82	1.6779 × 10^−15^

**Table 6:** GO cellular component enrichment analysis of intersecting targets shared by dapagliflozin and chronic kidney disease.

GO	Description	Number of Genes	Pathway Genes	Fold Enrichment	Enrichment FDR
GO:0005887	Integral component of plasma membrane	63	1894	3.49	1.8106 × 10^−16^
GO:0031226	Intrinsic component of plasma membrane	63	1978	3.34	7.9143 × 10^−16^
GO:0031982	Vesicle	97	4466	2.28	5.4624 × 10^−15^
GO:0031410	Cytoplasmic vesicle	73	2849	2.69	4.6323 × 10^−14^
GO:0097708	Intracellular vesicle	73	2851	2.69	4.6323 × 10^−14^
GO:0098590	Plasma membrane region	46	1332	3.62	1.4203 × 10^−12^
GO:0009986	Cell surface	40	1050	4.00	3.1974 × 10^−12^
GO:0101002	Ficolin-1-rich granule	20	223	9.41	3.1974 × 10^−12^
GO:0030141	Secretory granule	38	987	4.04	9.6672 × 10^−12^
GO:0070062	Extracellular exosome	58	2316	2.63	1.7271 × 10^−10^
GO:0005615	Extracellular space	75	3577	2.20	2.0066 × 10^−10^
GO:0043230	Extracellular organelle	58	2343	2.60	2.0066 × 10^−10^
GO:0065010	Extracellular membrane-bounded organelle	58	2343	2.60	2.0066 × 10^−10^
GO:1903561	Extracellular vesicle	58	2342	2.60	2.0066 × 10^−10^
GO:0099503	Secretory vesicle	39	1165	3.51	2.0319 × 10^−10^
GO:1904813	Ficolin-1-rich granule lumen	15	142	11.09	2.1738 × 10^−10^
GO:0030054	Junction	56	2293	2.56	7.1862 × 10^−10^
GO:0043235	Receptor complex	23	429	5.63	7.1862 × 10^−10^
GO:0005576	Extracellular region	87	4673	1.95	8.3852 × 10^−10^
GO:0045121	Membrane raft	20	351	5.98	4.7000 × 10^−9^

**Table 7:** Docking score and inhibition constant of dapagliflozin against the top 10 hub targets compared to the positive control.

No.	Protein	Drugs	Docking Score (kcal/mol)	Inhibition Constant (K_i_)
1	GAPDH	Heptelidic acid	−6.69	12.38 µM
	(PDB 6M61)	Dapagliflozin	−6.27	25.38 µM
2	IL6	HY-115910	−6.73	11.7 µM
	(PDB 1ALU)	Dapagliflozin	−6.83	9.87 µM
3	SRC	MPZ600	−8.98	262.77 nM
	(PDB 1Y57)	Dapagliflozin	−6.53	16.23 µM
4	EGFR	Erlotinib	−7.02	7.19 µM
	(PDB 1M17)	Dapagliflozin	−8.42	673.48 nM
5	HSP90AA1	N-benzyl-6-[(3-endo)-3-{[(3-methoxy-2-methylphenyl)carbonyl]amino}-8-azabicyclo[3.2.1]oct-8-yl]pyridine-3-carboxamide (592)	−13.86	69.38 pM
	(PDB 4AWQ)	Dapagliflozin	−8.5	587.42 nM
6	NFKB	BAY11-7082	−6.72	11.92 µM
	(PDB 3GUT)	Dapagliflozin	−6.23	27.3 µM
7	CASP3	(3S)-3-({[(5S,10aS)-2-{(2S)-4-carboxy-2-[(phenylacetyl)amino]butyl}-1,3-dioxo-2,3,5,7,8,9,10,10a-octahydro-1H-[1,2,4]triazolo[1,2-a]cinnolin-5-yl]carbonyl}amino)-4-oxopentanoic acid (B92)	−10.56	18.06 nM
	(PDB 3KJF)	Dapagliflozin	−7.46	3.39 µM
8	HSP0AB1	(5-fluoroisoindolin-2-yl)(4-hydroxy-5-isopropylbenzo[d]isoxazol-7-yl)methanone (KU3)	−8.29	841.21 nM
	(PDB 5UCJ)	Dapagliflozin	−6.51	16.83 µM
9	MAPK3	SCH772984	−13.06	266.89 pM
	(PDB 4QTB)	Dapagliflozin	−8.87	314.47 nM
10	GSK3B	5-[2,3-bis(fluoranyl)phenyl]-~{N}-[[1-(2-methoxyethyl)piperidin-4-yl]methyl]-1~{H}-indazole-3-carboxamide (N1Q)	−7.67	2.41 µM
	(PDB 6TCU)	Dapagliflozin	−7.70	2.27 µM

Criteria: strong inhibition when there is a docking score less than −8.0 kcal/mol and a docking score better than the positive control. GAPDH: glyceraldehyde-3-phosphate dehydrogenase, SRC: Proto-oncogene tyrosine-protein kinase Src, EGFR: epidermal growth factor receptor, HSP90AA1: heat shock protein HSP 90-alpha, CASP3: caspase-3, MAPK3: mitogen-activated protein kinase 3, GSK3B: glycogen synthase kinase-3 beta.

## Data Availability

The original contributions presented in this study are included in the article/Supplementary Material. Further inquiries can be directed to the corresponding author.

## Supplementary Materials

The following supporting information can be downloaded at: https://www.mdpi.com/article/10.3390/life15030437/s1, Table S1: Comparison of ADMET results: SwissADME vs. PkCSM.

## Abbreviations

The following abbreviations are used in this manuscript:

ADME	Absorption, distribution, metabolism, and excretion
ADMET	Absorption, distribution, metabolism, excretion, and toxicity
ADT	AutoDock Tools
AGE-RAGE	Advanced glycation end-products—Receptor for advanced glycation end-products
BBB	Blood–brain barrier
CASP3	Caspase-3
CKD	Chronic kidney disease
CYP	Cytochrome P450
DC	Degree centrality
EGFR	Epidermal growth factor receptor
eGFR	Estimated glomerular filtration rate
ESOL	Estimation of solubility
ESRD	End-stage renal disease
FDR	False discovery rate
GAPDH	Glyceraldehyde 3-phosphate dehydrogenase
GA	Genetic algorithm
GO	Gene ontology
GSK3β	Glycogen synthase kinase 3 beta
HBV	Hepatitis B virus
HF	Heart failure
HSP90AA1	Heat shock protein 90 alpha family class A member 1
HSP90AB1	Heat shock protein 90 alpha family class B member 1
IL-6	Interleukin-6
KEGG	Kyoto Encyclopedia of Genes and Genomes
LD50	Lethal dose, 50%
LOAEL	Lowest observed adverse effect level
MAPK	Mitogen-activated protein kinase
MAPK3	Mitogen-activated protein kinase 3
MAPK14	Mitogen-activated protein kinase 14
NFKB1	Nuclear factor kappa B subunit 1
Nrf2	Nuclear factor erythroid 2–related factor 2
PDB	Protein Data Bank
pkCSM	Pharmacokinetics of small molecules
PI3K-Akt	Phosphoinositide 3-kinase—Protein kinase B
PPI	Protein-protein interaction
SEA	Similarity ensemble approach
SMILES	Simplified molecular input line entry system
SRC	Proto-oncogene tyrosine-protein kinase Src
STRING	Search tool for the retrieval of interacting genes/proteins
SwissADME	Swiss absorption, distribution, metabolism, and excretion
TNF-α	Tumor necrosis factor-α
TPSA	Topological polar surface area
UniProt	Universal Protein Resource
SMILES	Simplified molecular input line entry system
