# Supplementary material for: Dapagliflozin in Chronic Kidney Disease: Insights from Network Pharmacology and Molecular Docking Simulation
Source: Life (Basel). 2025 Mar 11;15(3):437. doi: 10.3390/life15030437 (PMC11943942; doi:10.3390/life15030437)
Supplement: Supplementary file 1 [file life-15-00437-s001.zip › life-3502260-supplementary.pdf]

Dapagliflozin in Chronic Kidney Disease: Insights from Network Pharmacology and Molecular Docking Simulation

Atthaphong Phongphithakchai [1], Aman Tedasen [2,3], Ratana Netphakdee [2], Rattana Leelawattana [4], Thatsaphan Srithongkul [5], Sukit Raksasuk [5], Jason C Huang [6], Moragot Chatatikun [2,3*]

[1] Nephrology Unit, Division of Internal Medicine, Faculty of Medicine, Prince of Songkla University, Songkhla, Thailand; ton331@hotmail.com
[2] Department of Medical Technology, School of Allied Health Sciences, Walailak University, Nakhon Si Thammarat, Thailand; aman.te@wu.ac.th; ratana.ne@mail.wu.ac.th; moragot.ch@wu.ac.th
[3] Research Excellence Center for Innovation and Health Products (RECIHP), Walailak University, Nakhon Si Thammarat, Thailand; aman.te@wu.ac.th; moragot.ch@wu.ac.th
[4] Endocrinology and Metabolism Unit, Division of Internal Medicine, Faculty of Medicine, Prince of Songkla University, Songkhla, Thailand; lrattana@medicine.psu.ac.th
[5] Division of Nephrology, Department of Medicine, Faculty of Medicine, Siriraj Hospital, Mahidol University, Bangkok, Thailand; thatsaphan@gmail.com; kobsukit@gmail.com
[6] Department of Biotechnology and Laboratory Science in Medicine, National Yang Ming Chiao Tung University, Taipei, Taiwan; jasonhuang@nycu.edu.tw
* Correspondence: moragot.ch@wu.ac.th

ELECTRONIC SUPPLEMENTARY MATERIAL

Table S1. Comparison of ADMET results: SwissADME vs PkCSM.

Property	SwissADME	pkCSM
**#Aromatic heavy atoms**	12	N/A
**#H-bond acceptors**	6	N/A
**#H-bond donors**	4	N/A
**#Heavy atoms**	28	N/A
**#Rotatable bonds**	6	N/A
**Ali Class**	Moderately soluble	N/A
**Ali Log S**	-4.08	N/A
**Ali Solubility (mg/ml)**	3.43E-02	N/A
**Ali Solubility (mol/l)**	8.38E-05	N/A
**AMES toxicity**	N/A	No
**BBB permeability**	N/A	-0.997
**BBB permeant**	No	N/A
**Bioavailability Score**	0.55	N/A
**Brenk #alerts**	0	N/A
**Caco2 permeability**	N/A	0.938
**CNS permeability**	N/A	-3.34
**Consensus Log P**	2.18	N/A
**CYP1A2 inhibitior**	N/A	No
**CYP1A2 inhibitor**	No	N/A
**CYP2C19 inhibitior**	N/A	No
**CYP2C19 inhibitor**	No	N/A
**CYP2C9 inhibitior**	N/A	No
**CYP2C9 inhibitor**	No	N/A
**CYP2D6 inhibitior**	N/A	No
**CYP2D6 inhibitor**	Yes	N/A
**CYP2D6 substrate**	N/A	No
**CYP3A4 inhibitior**	N/A	No
**CYP3A4 inhibitor**	No	N/A
**CYP3A4 substrate**	N/A	No

**Egan #violations**	0	N/A
**ESOL Class**	Soluble	N/A
**ESOL Log S**	-3.78	N/A
**ESOL Solubility (mg/ml)**	6.84E-02	N/A
**ESOL Solubility (mol/l)**	1.67E-04	N/A
**Fraction Csp3**	0.43	N/A
**Fraction unbound (human)**	N/A	0.077
**Ghose #violations**	0	N/A
**GI absorption**	High	N/A
**Hepatotoxicity**	N/A	No
**hERG I inhibitor**	N/A	No
**hERG II inhibitor**	N/A	No
**iLOGP**	3.17	N/A
**Intestinal absorption (human)**	N/A	55.856
**Leadlikeness #violations**	1	N/A
**Lipinski #violations**	0	N/A
**log Kp (cm/s)**	-7.13	N/A
**Max. tolerated dose (human)**	N/A	0.507
**Minnow toxicity**	N/A	1.079
**MLOGP**	1.07	N/A
**MR**	104.82	N/A
**Muegge #violations**	0	N/A
**MW**	408.87	N/A
**Oral Rat Acute Toxicity (LD50)**	N/A	2.475
**Oral Rat Chronic Toxicity (LOAEL)**	N/A	3.63
**P-glycoprotein I inhibitor**	N/A	Yes
**P-glycoprotein II inhibitor**	N/A	Yes
**P-glycoprotein substrate**	N/A	Yes
**PAINS #alerts**	0	N/A
**Pgp substrate**	Yes	N/A

**Renal OCT2 substrate**	N/A	No
**Silicos-IT class**	Moderately soluble	N/A
**Silicos-IT Log P**	2.77	N/A
**Silicos-IT LogSw**	-4.46	N/A
**Silicos-IT Solubility (mg/ml)**	1.41E-02	N/A
**Silicos-IT Solubility (mol/l)**	3.44E-05	N/A
**Skin Permeability**	N/A	-2.751
**Skin Sensitisation**	N/A	No
**Synthetic Accessibility**	4.52	N/A
**T.Pyriformis toxicity**	N/A	0.289
**Total Clearance**	N/A	0.194
**TPSA**	99.38	N/A
**VDss (human)**	N/A	-0.331
**Veber #violations**	0	N/A
**Water solubility**	N/A	-3.433
**WLOGP**	1.52	N/A
**XLOGP3**	2.35	N/A
